# Bioimpedance spectroscopy for breast cancer-related lymphedema assessment: clinical practice guidelines

**DOI:** 10.1007/s10549-022-06850-7

**Published:** 2022-12-24

**Authors:** Chirag Shah, Pat Whitworth, Stephanie Valente, Graham S. Schwarz, Megan Kruse, Manpreet Kohli, Kirstyn Brownson, Laura Lawson, Beth Dupree, Frank A. Vicini

**Affiliations:** 1 Department of Radiation Oncology, Taussig Cancer Institute, Cleveland Clinic, Cleveland, OH USA; 2 Nashville Breast Center, Nashville, TN USA; 3 Department of Breast Surgery, Digestive Disease Institute, Cleveland Clinic, Cleveland, OH USA; 4 Deparment of Plastic Surgery, Dermatology and Plastic Surgery Institute, Cleveland Clinic, Cleveland, OH USA; 5 Department of Medical Oncology, Taussig Cancer Institute, Cleveland Clinic, Cleveland, OH USA; 6 Department of General Surgery, RWJ Barnabas Health, West Long Beach, NJ USA; 7 Department of General Surgery, University of Utah, Salt Lake City, UT USA; 8 Sedona, AZ USA; 9 Michigan Healthcare Professionals, Farmington Hills, MI USA

**Keywords:** Breast cancer, Lymphedema, Bioimpedance spectroscopy, L-Dex, Clinical guidelines

## Abstract

**Purpose:**

Breast cancer-related lymphedema (BCRL) represents a significant concern for patients following breast cancer treatment, and assessment for BCRL represents a key component of survivorship efforts. Growing data has demonstrated the benefits of early detection and treatment of BCRL. Traditional diagnostic modalities are less able to detect reversible subclinical BCRL while newer techniques such as bioimpedance spectroscopy (BIS) have shown the ability to detect subclinical BCRL, allowing for early intervention and low rates of chronic BCRL with level I evidence. We present updated clinical practice guidelines for BIS utilization to assess for BCRL.

**Methods and Results:**

Review of the literature identified a randomized controlled trial and other published data which form the basis for the recommendations made. The final results of the PREVENT trial, with 3-year follow-up, demonstrated an absolute reduction of 11.3% and relative reduction of 59% in chronic BCRL (through utilization of compression garment therapy) with BIS as compared to tape measurement. This is in keeping with real-world data demonstrating the effectiveness of BIS in a prospective surveillance model. For optimal outcomes patients should receive an initial pre-treatment measurement and subsequently be followed at a minimum quarterly for first 3 years then biannually for years 4–5, then annually as appropriate, consistent with previous guidelines; the target for intervention has been changed from a change in L-Dex of 10 to 6.5. The lack of pre-operative measure does not preclude inclusion in the prospective surveillance model of care.

**Conclusion:**

The updated clinical practice guidelines present a standardized approach for a prospective model of care using BIS for BCRL assessment and supported by evidence from a randomized controlled trial as well as real-world data.

**Supplementary Information:**

The online version contains supplementary material available at 10.1007/s10549-022-06850-7.

## Introduction

Breast cancer-related lymphedema (BCRL) represents a major potential sequela of breast cancer treatment and is a source of significant morbidity (limited limb mobility, infections) as well as quality of life detriment for patients, while also increasing costs for patients, payors, and the healthcare system [1, 2]. The risk of developing BCRL is based on the extent of locoregional therapy (axillary management, radiation therapy), systemic therapy (taxane chemotherapy, specifically docetaxel) as well as patient specific factors such as elevated BMI (> 30 kg/m^2^), with an incidence range of less than 10% for sentinel node biopsy alone to up to 50% for axillary dissection and adjuvant radiation therapy including regional nodal irradiation and systemic therapy [1, 3, 4]. Acknowledging the incidence and impact of BCRL, major international organizations including the American Cancer Society, American Society of Clinical Oncology (ASCO), the American Physical Therapy Association (APTA), the British Lymphology Society, the Australasian Lymphology Association, and the National Comprehensive Cancer Network (NCCN) have incorporated BCRL assessment and management as part of breast cancer survivorship and post-treatment surveillance guidelines (Table 1) [5–10].

**Table 1:** Clinical recommendations regarding breast cancer-related lymphedema

Organization	Recommendation
National Comprehensive Cancer Network Breast Cancer (2022) [7]	• “Lymphedema is a potential side effect after the treatment of axillary lymph node surgery resulting from damage to the lymphatic system. Early detection/diagnosis of lymphedema is key for optimal management. Consider pretreatment measurement of both arms as a baseline for patients with risk factors for lymphedema.” • “Educate, monitor, and refer for lymphedema management”
National Comprehensive Cancer Network Survivorship (2021) [8]	• “Lymphedema is a potential side effect after the treatment of cancer resulting from damage to the lymphatic system. Approximately three in four cases of lymphedema are diagnosed within three years of treatment; however, it can develop anytime in the life of the survivor. Depending on stage of diagnosis lymphoedema can be an acute or chronic condition.” • “Pretreatment limb measurement of both sides should be performed as a baseline for survivors with treatment-related or individual risk-factors, preferably by a trained lymphedema specialist” • “Early detection/diagnosis is key for optimal lymphedema management because stages 0 and 1 are reversible, whereas stages 2 and 3 are less responsive to treatment”
American Society of Clinical Oncology/American Cancer Society [5]	• “Counsel survivors on how to prevent/reduce the risk of lymphedema…” • “refer patients with clinical symptoms or swelling suggestive of lymphedema…”
American Physical Therapy Association (APTA) [6]	• Bioimpedance analysis (BIA) should be used to detect subclinical/early-stage lymphedema
British Lymphology Society [9]	• Those ‘at risk’ should be given information about what this means by a health care professional backed up with information leaflets (provided by the Lymphoedema Support Network (LSN) www.lymphoedema.org) or local leaflets. For individuals with cancer, the information should be provided before cancer treatment begins. A contact number of a key worker should be provided so a prompt referral can be made to a lymphoedema service if required. Ideally the key worker would be able to provide initial advice about managing lymphoedema symptoms and manage anxiety and expectations
Australasian Lymphology Association [10]	• Based on the currently available evidence, at this time, the ALA recommends that: • All patients be pre-operatively assessed using circumference (volume) measurements and/or bioimpedance spectroscopy • These measurements should be provided to the patient for ongoing monitoring where available/convenient • All patients should receive information about the possibility of developing lymphoedema as well as the early signs and symptoms and the known risk factors • Patients who are deemed to be at high risk for the development of breast cancer-related lymphedema should be monitored more regularly during the first year, and then at regular intervals for one more year • Patients who are deemed to be at lower risk for the development of breast cancer-related lymphoedema should be provided with information about who to contact if they have concerns about lymphoedema

Despite the growing acknowledgment of BCRL and its impact, standardized recommendations for BCRL surveillance with respect to diagnostic techniques, surveillance schedules, and intervention criteria for patients diagnosed with BCRL are not widely available. With respect to diagnostic techniques, growing data support early detection with subclinical BCRL allowing for early intervention [11]. Traditional BCRL diagnostic techniques such as tape measure have limited ability to detect subclinical BCRL, with newer techniques such as bioimpedance spectroscopy (BIS), 3D digital volumetry, ICG lymphography, and perometry able to detect subclinical BCRL [12, 13]. This ability to detect subclinical disease allows for early intervention with non-invasive strategies such as compression garments [13, 14]. This concept was validated in a prospective study from Stout-Gergich et al., which found that patients followed with perometry and treated with early intervention (compression garments) had reduced arm volumes and the need for further BCRL treatment [13, 14]. Similar findings have been seen with respect to BIS; data from the University of Pittsburgh, which incorporated BIS surveillance, found a reduction in clinical BCRL as compared to historical controls, which has also been confirmed in other series evaluating BIS [15–22]. With regards to surveillance schedules, current guidelines do not provide consistent surveillance schedules [5–10]. With regards to intervention criteria, criteria differ by diagnostic technique and within each technique there are differences, though modern trials are increasingly standardizing intervention criteria for arm volume assessments [23].

We have previously published clinical practice guidelines for utilizing BIS to assess for BCRL, which provided guidelines for BIS technique, patient population (all breast patients with targeted high risk population including those undergoing mastectomy, axillary dissection, greater than 6 sentinel nodes, regional nodal irradiation, or taxane chemotherapy), surveillance and screening schedules (prior to locoregional/neoadjuvant therapy, quarterly for 3 years, decrease year 4 and beyond as appropriate), and intervention criteria (L-Dex change of greater than 10) based on data available at the time [24]; however, since its publication, advances in BIS technology and additional data including the recent publication of a randomized controlled trial evaluating the technique as compared to tape measure have become available. For example, the original guidelines called for a change of greater than 10 in the L-Dex score to trigger intervention [12, 15, 24–27]. However, growing data supported a lower threshold for increased sensitivity to detect subclinical BCRL when going from three standard deviations (L-Dex change of 10) to two standard deviations (L-Dex change of 6.5) [12, 27–31]. Given such emerging data, we present updated clinical practice guidelines.

## Results

### Rationale for early detection and intervention

As noted above, the rationale for BCRL surveillance programs is data from previous studies that demonstrated a reduction in chronic BCRL with early intervention, coupled with current data showing subclinical BCRL detection and early non-invasive intervention reduced rates of chronic BCRL [32, 33]. Recently, a randomized trial evaluating BIS and early intervention was published; the PREVENT trial compared BCRL surveillance with BIS versus tape measure, with early intervention triggered by either evaluation method. Final results demonstrated that surveillance with BIS coupled with early intervention was associated with an 11.3% absolute reduction in complex decongestive physiotherapy (CDP), which per the study was defined as a surrogate for chronic BCRL [34]. Given the randomized nature, size of the study, and long-term follow-up, this recent trial provides level I evidence to support prospective surveillance with BIS in conjunction with early intervention to reduce chronic BCRL, something that is not available with other BCRL diagnostic techniques. Table 2 presents outcomes with prospective BCRL surveillance using BIS [15, 22, 35, 36].

**Table 2:** Outcomes with prospective BCRL surveillance with bioimpedance spectroscopy

	Study type	Number of patients	Screening frequency	Follow-up	Outcomes
PREVENT [34]	Randomized	879	Baseline, 3, 6, 12, 15, 18, 21, 24, 30, and 36 months	33 months	As compared to tape measure, BIS had reduced rates of CDP (19.2% vs. 7.9%)
University of Kansas Cancer Center [35]	Single-Institution	146	Baseline, 3, 6, 9, 1-,18, 24, 36, 48 month	21 months	Rate of persistent BCRL 6%
University of Pittsburgh [15]	Single-Institution	186	Baseline, every 3–6 months X 5 years	20 months (average)	Rate of clinical lymphedema 4.4% vs. 36.4% with control group
Macquarie University [16]	Single-Institution	753	Baseline or within 90 days of surgery		Reduced clinical lymphedema with BIS surveillance (14% vs. 39%), reduced severe lymphedema (Stage II-III, 4% vs. 24%)
Nashville Breast Center [17]	Single-Institution	505/93 (high risk)	–	24 months	High risk cohort (n = 93)- 11% required CDP, 3% at last follow-up had unresolved lymphedema
Breast Care Specialists [22]	Single-Institution	206	Pre-operative and post-operative measurements	26 months	23% elevated L-Dex score; no patients required CDP

### Clinical practice guidelines for bioimpedance spectroscopy in the management of breast cancer-related lymphedema

The updated clinical practice guidelines (Table 3) are based on the methods and data from the recent randomized trial and other real-world evidence [15–17, 23, 34, 35]. They are the consensus opinion of the authors based on clinical experience as well as a review of the literature.

**Table 3:** Clinical practice guidelines summary

	Recommendation
Who to screen?	Patients meeting at least one of the following criteria: 1. Axillary Management: Axillary Lymph Node Dissection or Sentinel Lymph Node Biopsy with > 6 nodes removed 2. Regional Node Irradiation 3. Taxane based chemotherapy 4. BMI > 30 kg/m^2^ 5. Mastectomy in the setting of invasive breast cancer
How often should patients have L-Dex measurements	Baseline Years 1–3: Quarterly Years 4–5: Semi-annually Years 6 +: Annually as clinically indicated
When to initiate BCRL treatment?	Change of L-Dex Score > 6.5 over baseline Compression garment × 4 weeks
Management Post Early-intervention	Measure following completion of intervention If remains abnormal, refer for complex decongestive physiotherapy For those returning to normal following intervention, follow quarterly for at least 3 years post-treatment

## Patient selection

Patient selection for BCRL surveillance should account for treatment and patient characteristics; while all patients are eligible to undergo BCRL surveillance, targeting those patients at highest risk to develop BCRL would improve the utility of surveillance. With respect to surveillance consideration, BIS should be considered for patients undergoing any surgical lymph node evaluation (sentinel lymph node biopsy alone, targeted axillary excision or axillary lymph node dissection), receiving regional node irradiation, and/or receiving taxane based chemotherapy; of note, while mastectomy was included as a criteria for the recent randomized trial and previous guidelines, patients undergoing mastectomy for prophylaxis or DCIS may not require BCRL surveillance, while those undergoing mastectomy for invasive malignancy should be considered [1, 3, 4, 24, 25, 37]. Additionally, patient characteristics that would suggest a role for surveillance include an elevated BMI (> 30 kg/m^2^) as well as rurality, which has been identified as a BCRL risk factor [1, 4, 34].

## BIS technique

**Fig. 1:**
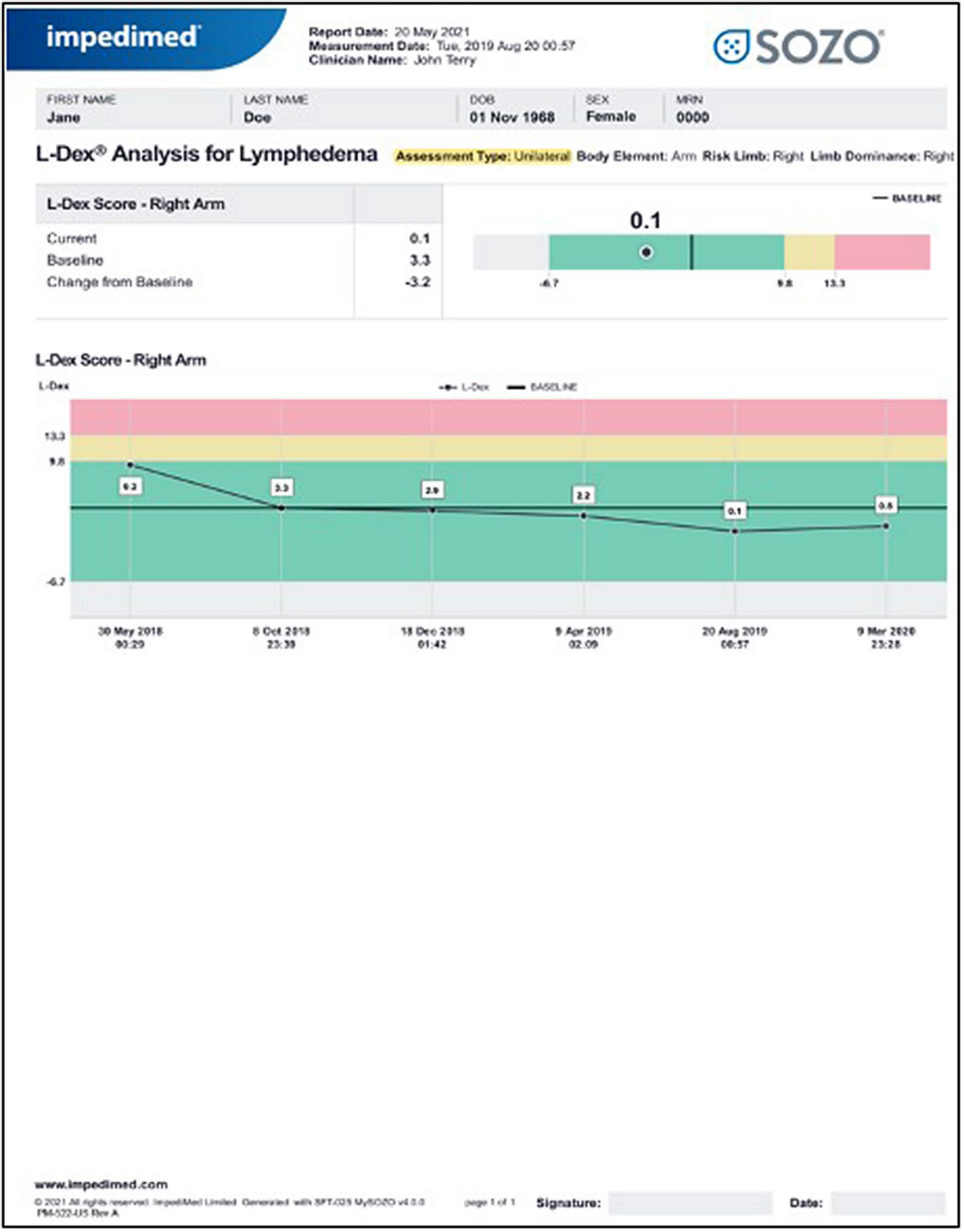
Reading from SOZO device

Previously, BIS guidelines based on the utilization the L-Dex U400 device were published; while BIS is the measurement, the L-Dex score has been utilized clinically and forms the basis for much of the data using BIS for BCRL assessment [24]. However, more recently, the SOZO device has been released simplifying BIS measurements, reducing test time, removing the need and cost associated with single use gel backed electrodes and eliminating the requirement for a dedicated procedure room to perform the test [38]. The SOZO device has been validated against the U400 and found to be substantially equivalent to the U400 by the FDA [38, 39]. The results are instantly displayed in numeric and graphical format enabling real-time review by the clinician (Fig. 1). Measurements should be performed by a trained health professional and may include a medical assistant or registered nurse, depending on clinic set up. Interpretation of results and initiation of treatment is commonly performed by a physician in the United States.

## Baseline measurement

Ideally, a pre-treatment baseline should be obtained to enable the earliest opportunity to intervene, but this may not be achievable for all patients. The lack of pre-treatment baseline measure does not preclude participation in a prospective BCRL surveillance program, consistent with previous guidelines as well as studies that have shown that early detection as part of a prospective surveillance program can be achieved in patients without pre-treatment baseline measurements when using bioimpedance [16, 24]. A baseline can be set for patients who present post-treatment with no clinical signs of lymphedema and an L-Dex score in the normal range and then followed for an increase of 6.5 of more (2 standard deviations) to initiate intervention [15, 16].

## Screening frequency

Determining the appropriate screening frequency with BIS is essential in implementing a BCRL surveillance program. Based on the recent randomized trial, L-Dex measurements should be taken at baseline (prior to locoregional or neoadjuvant therapy), 4–6 weeks after surgery (prior to initiation of radiation), and then subsequently at a minimum quarterly for the first 3 years post-treatment when the risk of developing lymphedema is the greatest [25, 40]. For years 4 and 5, assessments can be reduced to biannually then annually as appropriate [24]. These are consistent with previous guidelines and the recent randomized trial [24, 25]. While there has been some suggestion that shorter durations of surveillance may be appropriate, recent data has shown continued increases in L-Dex scores beyond 15 months suggesting the need for continued surveillance [1, 41]. At this time, there is no prospective data with long-term follow-up comparing alternative screening intervals.

## Intervention initiation

An important question facing clinicians utilizing BIS has been when to initiate early intervention with a compression garment; importantly, the goal of prospective BCRL surveillance is to risk stratify and identify those patients who can benefit from compression garment utilization rather than prescribing to all patients. When patients demonstrate an L-Dex increase of greater than 6.5, they should be prescribed a compression sleeve/sleeve and gauntlet delivering 20–30 mm of pressure for 4 weeks, 12 h per day; such an approach has been associated with reduced rates of clinical BCRL as compared traditional surveillance [16, 25]. Importantly, the compression garment and gauntlet should be appropriately fitted to avoid ill-fitting garments. Of note, despite concerns for increased triggers and unnecessary interventions with BIS surveillance, there were fewer triggers in the BIS group than the tape measure group seen in the recent randomized trial, highlighting the sensitivity and specificity of BIS, while allaying fears of overtreatment with BIS surveillance [34]. Following treatment with compression garment, repeat assessment should be performed and for those patients with persistent elevated score, referral for CDT should be considered. For those returning to normal, follow-up measurements should be performed quarterly for up to 3 years following breast cancer treatment. Of note, the recent RCT allowed patients who triggered an intervention to keep their garment and use as needed as part of the protocol, though this was only for patients who had already triggered.

## Discussion

BCRL represents one of the most feared complications for breast cancer survivors, impacting their quality of life as well as forcing patients to address the associated morbidities and costly, time-intensive interventions [42]. Given the increasing number of breast cancer survivors, assessing for BCRL as part of a prospective standardized survivorship plan is essential given that data supports the role of early detection and intervention in preventing chronic BCRL; for example, a recent meta-analysis found that prospective surveillance drastically reduced the rates of chronic BCRL [43]. To date, data support the utilization of BIS as part of a prospective model of care in which patients are followed closely at routine intervals that can result in early identification of lymphedema and improved patient outcomes [15, 35, 36]. Importantly, this approach is distinct from the treat all patients at risk of BCRL approach: a recent randomized study (which utilized BIS), found that compression for all patients undergoing ALND reduced rates of BCRL; however, this required all patients to undergo therapy rather than tailoring the need to for therapy to those demonstrating sub-clinical BCRL which may have an impact on psychosocial functioning, while also having short follow-up [44].

The present clinical guidelines present clinicians with a standardized evidence based approach to BCRL surveillance with follow-up time points as well as intervention criteria and methodology based on level I evidence. Importantly, the guidelines support that while the key to a successful BCRL prevention program is early identification and subsequent intervention, the absence of a pre-treatment baseline does not prohibit participation in prospective surveillance. The first visit can be considered baseline in patients who display no clinical signs of lymphedema, despite being post-treatment, allowing for BCRL surveillance for all at risk patients [15, 16]. Finally, it's important to recognize that these guidelines are for L-Dex utilization in prospective surveillance and do not address other measurement methods.

Implementing a prospective BCRL surveillance program will require multi-disciplinary collaboration with multiple models available. At some centers, BIS measurements are taken on all breast cancer patients in a multi-disciplinary clinic, with physicians of any discipline able to assess and initiate intervention with prescription/referral for compression garment; alternatively, some centers may use a similar model for intervention but limit assessments to patients with risk factors, which can be flagged in a chart/medical record. Alternatively, some practices may have a single discipline manage BCRL surveillance including breast surgeons, oncologists, or survivorship clinics; in such a model, the discipline can measure and prescribe/refer for intervention with measurements at each visit. Importantly, BIS is user friendly and any trained member of the health-care team can perform the actual measurement in a fraction of the time needed to perform tape measurements, with improved chronic BCRL outcomes with BIS.

A major concern with implementing BCRL surveillance using BIS are the associated costs. However, while acknowledging costs associated with prospective BCRL surveillance, it is important to recognize that the costs associated with chronic BCRL can include higher rates of hospitalizations as compared to breast cancer patients who do not develop BCRL as well as the costs of managing chronic BCRL [2, 45]. As such, studies have demonstrated the cost-effectiveness and value of prospective BCRL surveillance. Stout et al. evaluated the cost of prospective surveillance as compared to traditional care finding the cost to manage early BCRL was $636 per patient per year as compared to $3,125 with late-stage BCRL, offering the potential for a substantial cost savings [46]. For programs implementing BIS surveillance, a cost analysis based on the recent RCT demonstrated a substantial cost savings, regardless of program size, when implementing BIS prospective surveillance as compared to tape measure; use of BIS reduced costs by $356-$770 per patient and when accounting for potential hospitalizations by more than $16,000 at 1 year [47]. Together, these data support that prospective BCRL surveillance is a value-oriented approach, reducing chronic BCRL and the costs associated. Additionally, data from the recent RCT support that as compared to tape measure, BIS is cost effective, by reducing false positives, and tailoring early intervention to those patients at risk for chronic BCRL.

## Conclusion

Bioimpedance spectroscopy represents a standard diagnostic approach to assess for breast cancer-related lymphedema, allowing for early detection and treatment. BIS should be used as part of routine clinical care starting with measurement prior to treatment; however, BCRL surveillance can be utilized without a pre-treatment assessment. The updated clinical practice guidelines are supported by evidence from a randomized controlled trial and other real-world data.

## Supplementary Information

Below is the link to the electronic supplementary material.

Supplementary file1 (DOCX 20 kb)

## Data Availability

Enquiries about data availability should be directed to the authors.
