# Supplementary material for: Bioimpedance spectroscopy for breast cancer-related lymphedema assessment: clinical practice guidelines
Source: Breast Cancer Res Treat. 2022 Dec 24;198(1):1–9. doi: 10.1007/s10549-022-06850-7 (PMC9883343; doi:10.1007/s10549-022-06850-7)
Supplement: Supplementary file 1 — Supplementary file1 (DOCX 20 kb) [file 10549_2022_6850_MOESM1_ESM.docx]

**Supplemental information**

| **Supplemental Table 1: Comparisons of the MT and ONL thickness between neurotypical adults (NT) and autistic adults (ASD) on region and sector level.** | | | | | | | |
| --- | --- | --- | --- | --- | --- | --- | --- |
|  |  | **Arithmetic mean (SD)** | | **Results from the regression models** | | | |
| **Layer** | **Region** | **NT (N=31)** | **ASD (N=34)** | **𝛽_group_** | **SE** | ***p* value** | ***d*** |
| MT | Fovea | 284.08 (20.70) | 276.18 (22.62) | −8.84 | 5.51 | .114 (ns) | −0.41 |
|  | Parafovea | 1413.45 (58.58) | 1370.79 (56.11) | −46.14 | 14.24 | **.002 (*)** | −0.83 |
|  | Perifovea | 1233.29 (50.11) | 1203.32 (49.51) | −34.22 | 12.67 | **.009 (*)** | −0.69 |
| ONL | Fovea | 101.08 (9.47) | 95.31 (10.08) | −6.68 | 2.53 | **.010 (*)** | −0.68 |
|  | Parafovea | 318.81 (31.37) | 294.57 (32.14) | −26.21 | 8.27 | **.002 (*)** | −0.81 |
|  | Perifovea | 258.71 (26.7) | 239.78 (24.01) | −20.15 | 6.88 | **.005 (*)** | −0.75 |
| **Layer** | **Sector** | **NT (N=31)** | **ASD (N=34)** | **𝛽_group_** | **SE** | ***p* value** | ***d*** |
| MT | C0 | 284.08 (20.70) | 276.18 (22.62) | −8.84 | 5.51 | .114 (ns) | −0.41 |
|  | I1 | 354.52 (14.88) | 344.09 (13.33) | −11.15 | 3.61 | **.003 (*)** | −0.79 |
|  | I2 | 299.81 (11.14) | 292.41 (11.96) | −8.28 | 3.02 | **.008 (*)** | −0.70 |
|  | N1 | 357.64 (15.59) | 346.38 (15.94) | −12.13 | 3.85 | **.003 (*)** | −0.81 |
|  | N2 | 330.13 (15.95) | 319.88 (14.73) | −11.40 | 3.96 | **.005 (*)** | −0.74 |
|  | S1 | 357.23 (15.32) | 347.03 (14.19) | −10.84 | 3.73 | **.005 (*)** | −0.75 |
|  | S2 | 309.14 (13.10) | 304.07 (13.43) | −6.21 | 3.29 | .064 (ns) | −0.48 |
|  | T1 | 344.06 (14.43) | 333.29 (13.93) | −11.98 | 3.44 | **<.001 (*)** | −0.89 |
|  | T2 | 294.21 (11.91) | 286.96 (13.19) | −8.74 | 3.11 | **.007 (*)** | −0.72 |
| ONL | C0 | 101.08 (9.47) | 95.31 (10.08) | −6.68 | 2.53 | **.010 (*)** | −0.68 |
|  | I1 | 76.36 (7.02) | 70.24 (9.26) | −5.94 | 2.16 | **.008 (*)** | −0.70 |
|  | I2 | 59.47 (5.82) | 54.91 (5.55) | −4.82 | 1.55 | **.003 (*)** | −0.79 |
|  | N1 | 84.61 (8.62) | 78.81 (8.74) | −6.45 | 2.25 | **.006 (*)** | −0.73 |
|  | N2 | 66.27 (7.53) | 61.31 (6.81) | −5.47 | 1.93 | **.006 (*)** | −0.73 |
|  | S1 | 78.39 (9.23) | 72.85 (11.60) | −5.29 | 2.74 | .058 (ns) | −0.49 |
|  | S2 | 68.45 (7.46) | 64.28 (7.05) | −4.47 | 1.94 | **.025 (*)** | −0.59 |
|  | T1 | 79.45 (8.15) | 72.68 (8.31) | −7.18 | 2.14 | **.001 (*)** | −0.86 |
|  | T2 | 64.52 (6.70) | 59.28 (6.27) | −5.35 | 1.75 | **.003 (*)** | −0.79 |
| The arithmetic means (SD) and the results of the robust regression models are depicted for the three foveal regions and the single sectors of the ETDRS grid (37). Group coefficients (𝛽_group_) from the robust regression models (𝛽_0_= male NT), standard errors (SE), *p* values with adjusted significance level in parentheses (*/ns) and effects sizes (*d*) are depicted. Benjamini-Hochberg procedure (51) was applied for the region and sector analyses separately, in order to control the false discovery rate. The number of tests per analysis (region or sector level) was considered for the correction. Significant results are highlighted in bold.  Abbreviations: ASD = autism spectrum disorder; 𝛽_group_ = coefficient for the group parameter from the regression model; C0 = fovea; *d* = Cohen’s d; I = inferior, MT = macular thickness; N = nasal; NT = neurotypical adults; ns = not significant; ONL = outer nuclear layer; S = superior; SE = standard error of the coefficient; T = temporal; * = significant. | | | | | | | |
